# The Etiological Structure of Cognitive-Neurophysiological Impairments in ADHD in Adolescence and Young Adulthood

**DOI:** 10.1177/1087054718771191

**Published:** 2018-05-03

**Authors:** Giorgia Michelini, Celeste H. M. Cheung, Viryanaga Kitsune, Daniel Brandeis, Tobias Banaschewski, Gráinne McLoughlin, Philip Asherson, Frühling Rijsdijk, Jonna Kuntsi

**Affiliations:** 1 King’s College London, UK; 2 Heidelberg University, Mannheim, Germany; 3 University of Zurich, Switzerland

**Keywords:** ADHD, cognitive performance, event-related potentials, adolescence, adulthood, family study

## Abstract

**Objective:** Previous studies in children with ADHD identified two partially separable familial factors underlying cognitive dysfunction, but evidence in adolescents and adults is lacking. Here, we investigate the etiological structure of cognitive-neurophysiological impairments in ADHD in adolescents and young adults. **Method:** Factor analyses and multivariate familial models were run in 356 participants from ADHD and control sibling pairs aged 11 to 27 years on data on IQ, digit span forward (DSF) and backward (DSB), and cognitive-performance and event-related potential (ERP) measures from three cognitive tasks. **Results:** Three familial factors (cF_1-3_), showing substantial familial overlap with ADHD, captured the familial covariation of ADHD with nine cognitive-ERP measures. cF_1_ loaded on IQ, mean reaction time (MRT), and reaction-time variability (RTV); cF_2_ on DSF and DSB; and cF_3_ on number of errors and ERPs of inhibition and error processing. **Conclusion:** These results identify three partially separable etiological pathways leading to cognitive-neurophysiological impairments in adolescent and adult ADHD.

## Introduction

The majority of children clinically diagnosed with ADHD continue to meet ADHD diagnostic criteria in full or in partial remission in adolescence and adulthood (Biederman et al., 2009; Cheung et al., 2015; Faraone, Biederman, & Mick, 2006; Karam et al., 2015; van Lieshout et al., 2016). In addition to symptoms of inattention and hyperactivity-impulsivity, adolescents and adults with ADHD typically show the same wide range of impairments in multiple cognitive functions that also characterize children with this disorder (Cheung et al., 2016; Hervey, Epstein, & Curry, 2004; Kuntsi et al., 2010; Uebel et al., 2010). For example, deficits in executive processes, such as inhibition and working memory, and in non-executive processes, such as preparation-vigilance impairments, have been found in individuals with ADHD in adolescence and adulthood (Cheung et al., 2016; Hervey et al., 2004; Mostert et al., 2015). The investigation of brain activity during cognitive tasks has further revealed widespread neurophysiological impairments, such as atypical brain activity during error processing, attentional allocation, and response inhibition, in adolescents and adults with ADHD (Cheung et al., 2016; Groom et al., 2010; McLoughlin et al., 2009; Michelini, Kitsune, Hosang, et al., 2016; Woltering, Liu, Rokeach, & Tannock, 2013). The evidence of multiple cognitive and brain abnormalities in ADHD has contributed to a shift in the theoretical understanding of the disorder: from models that propose the existence of a single deficit, for example in inhibition (Barkley, 1997), as responsible for the multiple cognitive impairments, to models that argue for multiple underlying factors and pathways to ADHD (Castellanos & Proal, 2012; Halperin & Schulz, 2006; Johnson, 2012).

Twin and family studies have consistently reported high genetic/familial influences and limited-to-no role of the shared environment on ADHD (Burt, Larsson, Lichtenstein, & Klump, 2012; Faraone et al., 2005). In childhood, the genetic/familial influences on ADHD also show strong overlap with those on cognitive impairments (Andreou et al., 2007; Frazier-Wood et al., 2012; Kuntsi et al., 2010; Wood, Asherson, van der Meere, & Kuntsi, 2010; Wood et al., 2011). Sibling studies have revealed two partially separable familial factors underlying the structure of cognitive impairments in ADHD in children, one capturing reaction-time variability (RTV) and another capturing executive function, such as response accuracy (Kuntsi et al., 2010) and working memory (Frazier-Wood et al., 2012). IQ may also represent a separate process, as the genetic/familial effects that ADHD shares with IQ are largely separate from those that ADHD shares with other cognitive impairments (Rommelse et al., 2008; Wood et al., 2010; Wood et al., 2011). Although ADHD persists in a significant number of individuals diagnosed in childhood, the extent to which these cognitive impairments show a similar etiological structure and share familial influences with ADHD in adolescents and adults is still unknown.

In our recent follow-up study of adolescents and young adults with a childhood combined-type ADHD diagnosis, we found a separation between impairments in cognitive and brain function processes in relation to ADHD outcomes (persistence/remission) at follow up. Cognitive and neurophysiological measures of preparation-vigilance processes (e.g., RTV, omission errors [OE], event-related potentials [ERPs] of response preparation), error detection, and IQ were uniquely linked to ADHD persistence/remission at follow up (Cheung et al., 2016; James et al., 2017; Michelini, Kitsune, Cheung, et al., 2016), as individuals with persistent ADHD, but not with remitted ADHD, showed impairments in these measures. IQ in childhood further predicted ADHD persistence/remission, suggesting that IQ may represent a moderator of outcome (Cheung et al., 2015). In contrast, executive function measures (e.g., working memory and inhibition), despite being sensitive to impairments in ADHD persisters, were unrelated to ADHD outcome, as individuals with persistent and remitted ADHD were indistinguishable on these measures (Cheung et al., 2016; Michelini, Kitsune, Cheung, et al., 2016). Overall, we proposed that, in adolescents and young adults with ADHD, cognitive-neurophysiological impairments may reflect three processes: (a) markers of persistence/remission (e.g., preparation-vigilance measures), (b) processes that are not associated with ADHD outcome (executive function), and (c) potential moderators of ADHD outcome (IQ) (Cheung et al., 2016). All three processes were impaired in adolescents and adults with persistent ADHD, suggesting a possible phenotypic separation of impairments in these three cognitive-neurophysiological processes in persistent ADHD. Yet, it remains unclear whether one or multiple etiological factors underlie the association between such impairments and the disorder, as no study to date has examined the etiology of multiple cognitive and brain impairments in adolescent and adult ADHD.

The present study aims to investigate, for the first time, the etiological structure underlying cognitive-neurophysiological processes in ADHD in adolescence and early adulthood, in our follow up of individuals from ADHD and control sibling pairs initially assessed in childhood (Andreou et al., 2007; Kuntsi et al., 2010; Wood, Asherson, Rijsdijk, & Kuntsi, 2009; Wood et al., 2011). In previous analyses at follow up, we found a broad range of impairments in cognitive and brain functions in individuals with persistent ADHD compared to controls (Cheung et al., 2017; Cheung et al., 2016; Michelini, Kitsune, Cheung, et al., 2016). Here, we aim to take the most comprehensive approach to date in examining whether one or multiple etiological processes underlie such impairments with persistent ADHD in this age group. We predict that, in line with studies on cognitive impairments in children (Frazier-Wood et al., 2012; Kuntsi et al., 2010; Wood et al., 2011), multiple and partially separable etiological processes would account for the presence of impairments in cognitive and brain function in the disorder.

## Method and Materials

### Sample

The sample consisted of 404 participants, including 226 participants from ADHD sibling pairs (each including one *Diagnostic and Statistical Manual of Mental Disorders* [4th ed.; *DSM IV*; American Psychiatric Association, 1994] ADHD proband and one affected or unaffected sibling) and 178 participants from control sibling pairs (both without ADHD) who had taken part in our previous research (Chen et al., 2008; Kuntsi et al., 2010) (see Supplementary material). At initial assessment (age range = 6-17 years, *M* = 11.80, standard deviation [*SD*] = 2.96), ADHD participants recruited from specialist clinics and their closest-age siblings were invited to participate. Control participants were recruited from schools. At follow up, which took place on average 5.8 years (*SD* = 1.24) after the childhood assessment, 30 childhood ADHD probands were excluded for no longer meeting *DSM-IV* ADHD criteria (*n* = 25), not having combined-type ADHD in childhood (*n* = 3), or due to EEG equipment failure (*n* = 2). Nine siblings of ADHD probands were excluded as they were unaffected in childhood but met *DSM-IV* ADHD criteria at follow up (*n* = 3) or their diagnostic status could not be determined due to missing parent-reported data on impairment (*n* = 6). Nine controls were excluded due to meeting ADHD criteria at follow up based on parent-reported ADHD ratings (Barkley Informant Rating Scale; Barkley & Murphy, 2006). The final sample for analyses consisted of 87 individuals with persistent ADHD and 100 unaffected siblings (69 full pairs, 49 singletons), and 169 control siblings (76 full pairs, 17 singletons; Table 1). Among participants with persistent ADHD, 60% (*n* = 52) met criteria for the combined subtype, 32% (*n* = 28) met criteria for predominantly inattentive subtype, and 8% (*n* = 7) met criteria for predominantly hyperactivity-impulsivity subtype at follow up. Written informed consent was obtained from all participants and the study was approved by the London-Surrey Borders Research Ethics Committee (NRES 09/H0806/58).

**Table 1.** Sample Demographic Information Divided by Group, With Test for Statistical Difference.

	ADHD probands (*n* = 87)	Unaffected siblings (*n* = 100)	Controls (*n* = 169)	*p*	ADHD probands vs. controls (*p*)	ADHD probands vs. unaffected siblings (*p*)	Unaffected siblings vs. controls (*p*)
Sex (M: F)	72:15	43:57	129:40	**<.001**	.21	**<.001**	**<.001**
Age	18.31 (3.03)	18.56 (3.33)	17.75 (2.17)	.08	.14	.53	**.03**

*Note.* Significant differences are indicated in bold. Group differences on sex were tested via chi-square test; group differences on other measures were tested with regression models. Group differences between ADHD and control participants were reported in previous analyses on this sample (Cheung et al., 2016; Michelini, Kitsune, Cheung, et al., 2016). F = female; M = male.

### ADHD Diagnosis

ADHD diagnostic status in ADHD sibling pairs was assessed with the Diagnostic Interview for ADHD in Adults (DIVA; Ramos-Quiroga et al., 2019), a semistructured interview designed to evaluate the *DSM-IV* criteria for childhood and adult ADHD. Evidence of impairment commonly associated with ADHD was assessed with the Barkley’s Functional Impairment Scale (BFIS; Barkley & Murphy, 2006), by trained researchers, along with the DIVA during face-to-face interviews with parents. A separate interview was conducted with the ADHD probands and their siblings. Parent-report DIVA and impairments were used to determine ADHD status based on *DSM-IV*, as these were validated against objective markers (cognitive-performance and EEG measures) in this sample, whereas the same objective markers showed limited agreement with self-reported ADHD (Du Rietz et al., 2016). ADHD symptoms were assessed in control participants using the parent-rated Barkley Informant Rating Scale (Barkley & Murphy, 2006).

### Procedure

Participants attended a single 4-hr research session (including breaks) for IQ, digit span, and cognitive-EEG assessments (Table 2). For each sibling pair in both ADHD and control groups, one of the siblings was administered the IQ and digit span assessment, followed by a battery of three cognitive-EEG tasks, and vice versa for the other sibling. This was counterbalanced by proband-sibling group. The three tasks in the cognitive-EEG battery were administered in the same order. For those prescribed stimulants (*n* = 52), a 48-hr ADHD medication-free period was required prior to cognitive-EEG assessments.

**Table 2.** Short Description of the Tasks Included in the Cognitive Assessment and Cognitive-EEG Battery.

Task name	Description	Measures extracted from the task	Measures included in analyses
WASI (Wechsler, 1999)	The vocabulary and block design subtests were used to derive an estimate of IQ	Total IQ	Total IQ
Digit span subtest from WISC-III (Wechsler, 1991), or the WAIS-III (Wechsler, 1997)	The digit span subtest from the WISC-III or the WAIS-III was administered to participants aged below 16 and aged 16 or above, respectively, to obtain DSF and DSB. The forward test measures verbal short-term memory, while the backward test requires is a measure of working memory	DSF, DSB	DSF, DSB
CPT-OX (Doehnert et al., 2008, Valko et al., 2009**)**	The CPT-OX task consists of 400 letter arrays formed of a center letter with incompatible flankers on each side, and probes attention, preparation, and response inhibition. Stimuli are presented for 150 ms with an stimulus onset asynchrony of 1.65 s in a pseudorandomized order at the center of a computer monitor. The task involves the presentation of 80 Cues ("XOX") followed either by 40 Go ("OXO") and 40 NoGo ("XDX") stimuli, alternated with random letter arrays as distractors. Participants are instructed to respond only to Cue-Go sequences, and to withhold the response in presence of a NoGo stimulus, of a Go not preceded by a Cue (40 trials), or of any other irrelevant letters. Task duration was approximately 11 min	*Cognitive performance*: MRT, RTV (i.e., *SD* of reaction times), CE (i.e., response to NoGo), OE (i.e., nonresponse to Go). *ERPs*: Cue-P3 (attentional orienting), CNV (response preparation), and NoGo-P3 (response inhibition)	OE, NoGo-P3
Eriksen Arrow Flanker Task (Albrecht et al., 2008, McLoughlin et al., 2009)	This performance monitoring task is an adaptation of the Eriksen Flanker paradigm designed to increase cognitive load for adults. In each trial, a central black fixation mark is replaced by a target arrow. Participants are instructed to indicate whether this arrow points toward the left or right by pressing corresponding response buttons with their left or right index fingers. Two flanker arrows identical in shape and size to the target appear above and below the center of the target arrow 100 ms prior to each target arrow. Both flankers either point in the same (congruent) or opposite (incongruent) direction to the target. Conflict monitoring is maximal during the incongruent condition. When the target appears, both target and flankers remain on the screen for a further 150 ms, with a new trial being presented every 1.65 s. Trials were arranged in 10 blocks of 40 trials. Task duration was approximately 13 min	*Cognitive performance*: MRT, RTV, and number of errors (left-right errors occurring when participants chose the wrong left or right response); all obtained separately both for congruent and incongruent conditions (congruent MRT, congruent RTV, CongE; incongruent MRT, incongruent RTV, IncongE). *ERPs*: N2 (conflict monitoring), ERN (automatic error processing), and Pe (conscious error processing), all obtained from the incongruent condition of this task only	CongE, ERN
Fast Task (Andreou et al., 2007)	This is a four-choice reaction-time task probing attentional processes. In a baseline (slow, unrewarded) condition (72 trials), four empty circles (warning signals, arranged horizontally) first appear for 8 s, after which one of them (the target) is colored in. Participants are instructed to press the response key that directly corresponds to the position of the target. Following a response, the stimuli disappear from the screen and a fixed intertrial interval of 2.5 s follows. Speed and accuracy are emphasized equally. If the participant does not respond within 10 s, the trial terminates. The baseline condition lasts approximately 15 min. A comparison condition (80 trials) with a fast event rate (1 s) and incentives follows the baseline condition. The fast-incentive condition lasts approximately 5 min	*Cognitive performance*: MRT, RTV from the baseline condition (more sensitive to ADHD [Kuntsi et al., 2013, Kuntsi & Klein, 2012]). *ERPs*: P3 (attentional orienting) from the baseline condition	MRT, RTV

*Note.* EEG = Electroencephalography; WASI = Wechsler Abbreviated Scale of Intelligence; IQ = intelligence quotient; WISC-III = Wechsler Intelligence Scale for Children–III; WAIS-III = Wechsler Adult Intelligence Scale-III; DSF = digit span forward; DSB = digit span backward; CPT-OX = Cued Continuous Performance Test; MRT = mean reaction time; RTV = reaction time variability; CE = commission errors; OE = omission errors; ERPs = event-related potentials; Cue-P3 = P3 amplitude in the Cue condition; CNV = contingent negative variation; NoGo-P3 = P3 amplitude in the NoGo condition; CongE = conguent errors; IncongE = incongruent errors; ERN = error-related negarivity; Pe = error positivity.

### Electrophysiological Recording and Analysis

The EEG was recorded from a 62 channel DC-coupled recording system (extended 10-20 montage), using a 500 Hz sampling rate, impedances under 10 kΩ, and FCz as the recording reference. The electro-oculograms (EOGs) were recorded from electrodes above and below the left eye and at the outer canthi. EEG data were analyzed using Brain Vision Analyzer 2.0 (Brain Products, Germany). Raw EEG recordings were down-sampled to 256 Hz, re-referenced to the average of all electrodes, and filtered using Butterworth band-pass filters (0.1-30 Hz, 24 dB/oct). Electrical or movement artifacts were removed following visual inspection. Ocular artifacts were corrected using the infomax Independent Component Analysis (ICA) algorithm (Jung et al., 2000). Sections of data containing artifacts exceeding ±100 µV or with a voltage step >50 µV were automatically rejected. ERPs were extracted from the CPT-OX (Cue-P3, CNV, NoGo-P3), arrow flanker task (N2, ERN, Pe in the incongruent condition), and Fast task (P3 in the baseline condition) following procedures used in previous analyses on this sample (Cheung et al., 2017; Cheung et al., 2016; Michelini, Kitsune, Cheung, et al., 2016) (Supplementary material).

### Statistical Analyses

#### Multivariate sibling-data model fitting

Sibling-data model fitting was accomplished by structural equation model fitting analyses (SEM) using the OpenMx package in R (Boker et al., 2011). As siblings share on average 50% of their segregating genes and 100% of the common environment, we can decompose the variance/covariance of traits into contributions of familial influences (the combined effects of shared genetic and shared environmental effects) and nonfamilial influences (individual-specific effects and possible measurement error; Cheung et al., 2012; Kuntsi et al., 2010). Sibling-pair data allow us to derive: phenotypic correlations in each sibling, for example, correlation between IQ and ADHD, constrained across birth order; cross-sibling/within-trait correlations, for example, correlation between Sibling 1 and Sibling 2 for IQ; and cross-sibling/cross-trait correlations, constrained such that, for example, correlations between IQ in Sibling 1 and ADHD in Sibling 2 equals the correlation of IQ in Sibling 2 and ADHD in Sibling 1. The cross-sibling/within-trait and the cross-sibling/cross-trait correlations allow to estimate, respectively, the familial variance of a trait and the familial overlap between traits. Given the selected nature of this sample (selection of ADHD probands), ADHD status was included in all models with its parameters fixed to population-known values, based on previous evidence and consistent with our previous work (Cheung et al., 2012; Frazier-Wood et al., 2012; James, Cheung, Rijsdijk, Asherson, & Kuntsi, 2016; Kuntsi et al., 2010): the cross-sibling/within-trait correlation (correlation between siblings in each pair) was fixed to 0.40 (Chang, Lichtenstein, Asherson, & Larsson, 2013; Larsson, Chang, D’Onofrio, & Lichtenstein, 2014); the familiality to 0.40 (representing 80% genetic variance in case of null shared environmental effects; Larsson et al., 2013); and prevalence of 5% (Willcutt, 2012; *z* score set at 1.64). For further explanation of this approach, see Supplementary material and Rijsdijk et al. (2005). A liability threshold model framework, which assumes that the liability of ADHD is underpinned by a normally distributed continuum of risk (Rijsdijk & Sham, 2002; Rijsdijk et al., 2005), was used to account for the fact that ADHD was measured as present/absent. Model-fitting analyses were performed with raw data maximum likelihood estimation incorporating all available data points (thus allowing no listwise/pairwise deletion when data in sibling pairs were missing).

#### Preliminary analyses and variable selection

Preliminary constrained correlation bivariate models between ADHD and 22 cognitive-ERP variables extracted from our large cognitive-neurophysiological battery (sensitive to ADHD-control differences in this sample; Cheung et al., 2017; Cheung et al., 2016; Michelini, Kitsune, Cheung, et al., 2016) were carried out to reduce the number of variables included in multivariate models. This variable selection step was necessary due to the limit in the number of variables that can be included in multivariate SEM (Kuntsi et al., 2010; Loken, Hettema, Aggen, & Kendler, 2014). A phenotypical association with ADHD and an evidence of familial effects are prerequisites to any familial overlap between two variables. As such, cognitive-ERP variables were only included if they had (a) a phenotypic correlation with ADHD above the threshold of 0.20, corresponding to modest-to-large effect sizes (Cohen, 1988), and (b) significant cross-sibling/within-trait correlations, indicating similarity between siblings (Table S1). Following preliminary analyses, nine variables were included with ADHD status in multivariate models (Table S2): IQ, digit span forward (DSF), digit span backward (DSB); ERN (Figure S2), and congruent errors (CongE) from the arrow flanker task; NoGo-P3 (Figure S3) and OE from the CPT-OX; and mean reaction time (MRT) and RTV from the Fast task (baseline condition). ERN, NoGo-P3, and MRT were transformed to normality using the square root transformation, while RTV was log-transformed. IQ, DSF, and DSB residuals were normally distributed. These measures were included as continuous variables. OE and CongE were highly skewed and could not be normalized using any transformation methods. They were therefore modeled as ordinal using 3 and 4 equal-sized categories, respectively. Age and sex were controlled for in all analyses as is standard practice for family model-fitting studies (McGue & Bouchard, 1984), by regressing out age and sex effects from continuous variables (before transforming to normality) and estimating age and sex effects on the mean for ordinal variables.

#### Cholesky and factor models

**Figure 1.**
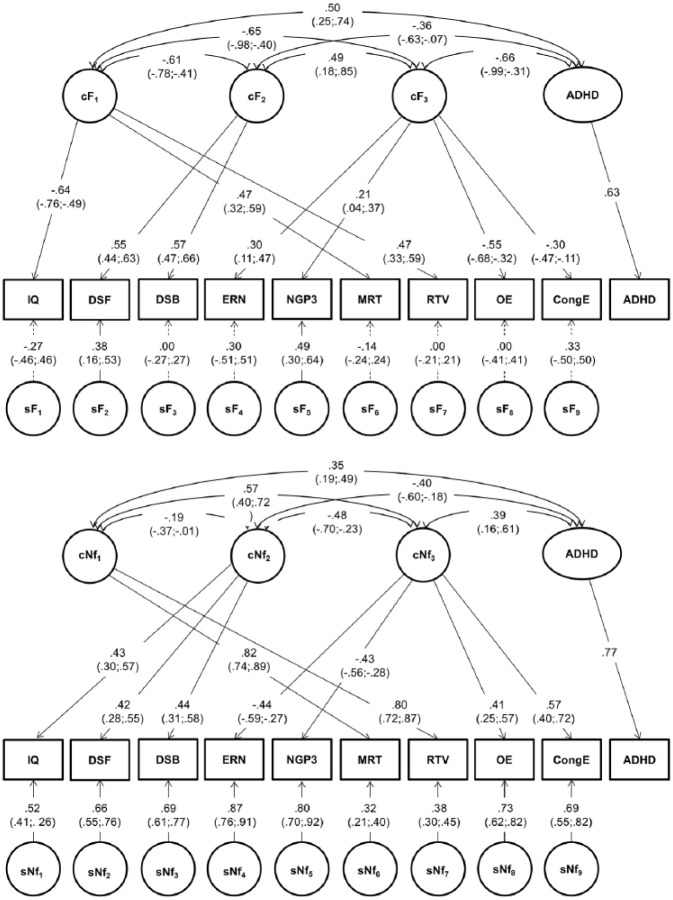
Confirmatory Factor model between cognitive-ERP variables and ADHD. *Note.* Significant parameters are indicated with solid lines (*p* < .05) and nonsignificant parameters with dotted lines. ERP = event-related potential; cF_1-3_ = common familial factors; cNf_1-3_ = common nonfamilial factors; IQ = intelligence quotient; DSF = digit span forward; DSB = digit span backward; ERN = error-related negativity amplitude from the flanker task; NoGo-P3 = P3 amplitude in the NoGo condition from the cued continuous performance test; MRT = mean reaction time from the fast task; RTV = reaction time variability from the fast task; OE = omission errors from the cued continuous performance test; CongE = errors in the congruent condition of the flanker task; sF_1-9_ = variable-specific familial influences; sNf_1-9_ = variable-specific nonfamilial influences.

A multivariate Cholesky decomposition (Rijsdijk & Sham, 2002) was used to decompose the variance/covariance structure of the cognitive-ERP variables and ADHD into familial and nonfamilial influences. The correlated factors solution of this decomposition yielded familial and nonfamilial correlation matrices between all variables, which provide the degree of overlap between etiological influences between two variables at a time (e.g., IQ and ADHD). We examined the etiological factor structure underlying cognitive-ERP variables and ADHD in a more parsimonious model, following a two-step approach employed in previous work (Frazier-Wood et al., 2012; Kuntsi et al., 2010). First, the derived familial and nonfamilial correlations between the nine cognitive-ERP variables were used as input to two separate exploratory factor analyses (EFAs) in R to extract the factor structure (Supplementary material). Separate EFAs were carried out on the familial and nonfamilial correlations between the nine cognitive-ERP variables. This allowed detection of possible differences between the familial and nonfamilial effects on the number of extracted factors or in how they load on variables. Three factors with an eigenvalue >1 were identified in both EFAs (Figure S1). Each factor explained >10% of the total variance in either EFA (Table S3). As cognitive-ERP variables mapped onto cognitive processes which are likely to be interrelated (Jewsbury, Bowden, & Strauss, 2016; Kovas & Plomin, 2006), we allowed the extracted factors to correlate by applying an oblique (oblimin) rotation (Gerbing & Hamilton, 1996; Widaman, 1993). Second, we specified the three factors, their correlations, and loadings (Table S3) separately for familial and nonfamilial influences in a confirmatory three-factor model, including also ADHD, using OpenMx. Familial and nonfamilial paths from the extracted common factors were specified for each variable from the factor with strongest loading in the EFAs, while ADHD and its fixed familial and nonfamilial influences were modeled separately (Figure 1). Familial common paths on DSF and DSB, as well as nonfamilial common paths on MRT and RTV, were constrained to be equal for model identification purposes. Correlation paths were specified among each factor loading on cognitive-ERP measures and ADHD. The residual variance of cognitive-ERP variables not accounted for by common factors was measured by variable-specific familial and nonfamilial paths. For comparisons with other models see Table S4.

## Results

### Phenotypic Correlations

Phenotypic correlations between cognitive-ERP variables and ADHD were all significant, with positive correlations ranging from .17 to .88 and negative correlations from −.19 to −.39 (Table 3). The only nonsignificant correlation was between ERN and DSF (*r*_Ph_ = .12, CIs [−0.01, 0.27]). ADHD showed moderate negative correlations with IQ, DSF, DSB, ERN, NoGo-P3, and moderate positive correlations with MRT, RTV, OE, and CongE.

**Table 3.** Phenotypic, Familial, and Nonfamilial Correlations Between Study Variables.

	IQ	DSF	DSB	ERN	NGP3	MRT	RTV	OE	CongE	ADHD
Phenotypic correlations										
IQ	1									
DSF	**.40** **[.30, .50]**	1								
DSB	**0.41** **[.32, .51]**	**.51** **[.43, .58]**	1							
ERN	**.16** **[.04, .28]**	.12 [−.00, .27]	**.17** **[.05, .27]**	**1**						
NoGo-P3	**.13** **[.02, .25]**	**.20** **[.08, .28]**	**.18** **[.07, .31]**	**.23** **[.13, .34]**	1					
MRT	**−.38** **[−.47, −.27]**	**−.19** **[−.30, −.07]**	**−.23** **[−.33, −.11]**	**−.24** **[−.35, −.13]**	**−.28** **[−.38, −.17]**	1				
RTV	**−.36** **[−.45, −.26]**	**−.16** **[−.28, −.05]**	**−.24** **[−.35, −.13]**	**−.30** **[−.38, −.18]**	**−.23** **[−.34, −.12]**	**.88** **[.85, .89]**	1			
OE	**−.33** **[−.44, −.20]**	**−.18** **[−.29, −.05]**	**−.25** **[−.37, −.12]**	**−.36** **[−.45, −.22]**	**−.31** **[−.42, −.20]**	**.35** **[.23, .46]**	**.37** **[.23, .53]**	1		
CongE	**−.21** **[−.33, −.10]**	**−.26** **[−.37, −.14]**	**−.20** **[−.32, −.08]**	**−.39** **[−.49, −.27]**	**−.30** **[−.42, −.18]**	**.32** **[.20, .43]**	**.33** **[.23, .45]**	**.37** **[.21, .48]**	1	
ADHD	**−.37** **[−.46, −.25]**	**−.21** **[−.32, −.08]**	**−.27** **[−.39, −.15]**	**−.24** **[−.37, −.10]**	**−.25** **[−.36, −.13]**	**.33** **[.20, .44]**	**.42** **[.29, .52]**	**.34** **[.20, .46]**	**.32** **[.18, .44]**	1
Familial correlations										
IQ	**1**									
DSF	**.50** **[.04, .82]**	**1**								
DSB	**.58** **[.32, .81]**	**.83** **[.61, .98]**	1							
ERN	.31 [−.01, .28]	0.08 [−.36, .49]	.28 [−.18, .73]	1						
NoGo-P3	.17 [−.99, .53]	.27 [−.15, .63]	.25 [−.19, .70]	.12 [−.49, .59]	1					
MRT	**−.65** **[−.88, −.37]**	−0.29 [−.59, .05]	**−.60** **[−.90, −.24]**	**−.51** **[−.94, −.07]**	−.28 [−.65, .21]	1				
RTV	**−.66** **[−.94, −.41]**	−.26 [−.60, .10]	**−.56** **[−.90, −.16]**	**−.52** **[−.97, −.05]**	−.18 [−.61, .35]	**.95** **[.87, .99]**	1			
OE	**−.67** **[−.98, −.29]**	**−.41** **[−.82, −.01]**	**−.48** **[−.95, −.02]**	**−.72** **[−.97, −.18]**	−.36 [−.79, .25]	**.50** **[.02, .90]**	.49 [−.01, .91]	1		
CongE	−.25 [−.63, −.19]	−.23 [−.60, .27]	−.30 [−.77, .23]	**−.69** **[−.97, −.14]**	−.45 [−.84, .20]	.40 [−.18, .82]	**.52** **[.30, .91]**	**.48** **[.05, .94]**	1	
ADHD	**−.38** **[−.63, −.09]**	−.30 [−.60, .05]	**−.52** **[−.66, −.16]**	−.35 [−.83, .14]	−.37 [−.81, .09]	.33 [−.04, .69]	**.45** **[.07, .82]**	**.60** **[.13, .93]**	**.56** **[.06, .99]**	1
Nonfamilial correlations										
IQ	1									
DSF	**.31** **[.16, .45]**	1								
DSB	**.32** **[.16, .46]**	**.32** **[.17, .46]**	1							
ERN	.09 [−.06, .24]	.14 [−.02, .31]	.12 [−.04, .28]	1						
NoGo-P3	.12 [−.05, .28]	.16 [−.01, .32]	.15 [−.03, .31]	**.26** **[.10, .42]**	1					
MRT	**−.21** **[−.35, −.04]**	−.13 [−.29, .04]	−.05 [−.21, .12]	−.15 [−.31, .02]	**−.29** **[−.43, −.12]**	1				
RTV	**−.20** **[−.35, −.03]**	−.12 [−.29, .05]	−.11 [−.25, .05]	**−.22** **[−.37, −.06]**	**−.26** **[−.42, −.09]**	**.85** **[.80, .89]**	1			
OE	−.13 [−.33, .06]	−.06 [−.25, .14]	−.15 [−.33, .04]	**−.25** **[−.43, −.03]**	**−.28** **[−.47, −.10]**	**.30** **[.11, .47]**	**.36** **[.15, .51]**	1		
CongE	**−.20** **[−.38, −.04]**	**−.29** **[−.46, −.11]**	−.17 [−.33, .00]	**−.30** **[−.46, −.13]**	**−.24** **[−.43, −.07]**	**.30** **[.15, .47]**	**.30** **[.14, .45]**	**.30** **[.16, .52]**	1	
** **ADHD	**−.36** **[−.52, −.18]**	−.13 [−.31, .05]	−.14 [−.31, .05]	**−.20** **[−.38, −.01]**	**−.20** **[−.36, −.01]**	**.32** **[.14, .49]**	**.40** **[.22, .57]**	**.22** **[.03, .45]**	**.24** **[.02, .44]**	1

*Note.* Significant correlations are indicated in bold. IQ = intelligence quotient; DSF = digit span forward; DSB = digit span backward; ERN = error-related negativity amplitude from the flanker task; NoGo-P3 = P3 amplitude in the NoGo condition from the cued continuous performance test; MRT = mean reaction time from the fast task; RTV = reaction time variability from the fast task; OE = omission errors from the cued continuous performance test; CongE = errors in the congruent condition of the flanker task.

### Multivariate Cholesky Decomposition

Familial correlations of ADHD with IQ, DSB, RTV, OE, and CongE were significant and moderate-to-large, and moderate but nonsignificant with DSF, ERN, NoGo-P3 (Table 3). Nonfamilial correlations of ADHD with IQ, ERN, NoGo-P3, MRT, RTV, OE, and CongE were modest and significant, while the correlations of ADHD with DSF and DSB were small and nonsignificant.

### Multivariate Factor Model

Three familial and three nonfamilial factors emerged from EFAs, explaining the association between the nine cognitive-ERP variables (Figure S1). This factor structure informed the confirmatory factor model, which provided the best fit to the data (Table S4). The first familial factor (cF_1_) loaded onto IQ, MRT, and RTV; the second factor (cF_2_) onto DSF and DSB; and the third factor (cF_3_) onto ERN, NoGo-P3, OE, and CongE (Figure 1). The three familial factors accounted for most of the familial variance on cognitive-ERP measures, as variable-specific familial influences were in general low and nonsignificant, apart from those on DSF (0.13; 30% of familial variance) and NoGo-P3 (0.21; 81% of familial variance) (Table 4). The familial factors showed high intercorrelations and moderate-to-high correlations with familial influences on ADHD (Figure 1).

**Table 4.** Factor Structure and Standardized Familial and Nonfamilial Variance of Cognitive-ERP Measures, Also Split Up by Contribution of Each Factor and of Specific (Residual) Effects, With 95% Confidence Intervals in Brackets.

	Total	Common F1	Common F2	Common F3	Specific
Familial influences					
IQ	**.49 [.38, .59]**	**.41 [.24, .57]**			.08 [.00, .24]
DSF	**.43 [.31, .54]**		**.30 [.20, .40]**		**.13 [.02, .24]**
DSB	**.33 [.22, .43]**		**.33 [.22, .43]**		.00 [.00, .00]
ERN	**.17 [.02, .32]**			**.09 [.01, .22]**	.08 [.00, .21]
NoGo-P3	**.26 [.11, .40]**			**.05 [.01, .14]**	**.21 [.08, .34]**
MRT	**.24 [.12, .37]**	**.22 [.18, .34]**			.02 [.00, .06]
RTV	**.22 [.10, .35]**	**.22 [.10, .35]**			.00 [.00, .00]
OE	**.30 [.10, .45]**			**.30 [.10, .47]**	.00 [.00, .00]
CongE	**.20 [.05, .39]**			**.09 [.01, .22]**	.11 [.00, .25]
Nonfamilial influences					
IQ	**.51 [.41, .62]**		**.19 [.09, .33]**		**.32 [.19, .30]**
DSF	**.57 [.46, .69]**		**.17 [.08, .30]**		**.40 [.27, .52]**
DSB	**.68 [.57, .78]**		**.20 [.09, .33]**		**.48 [.37, .58]**
ERN	**.83 [.68, .98]**			**.19 [.08, .31]**	**.64 [.49, .80]**
NoGo-P3	**.74 [.61, .89]**			**.18 [.08, .31]**	**.56 [.42, .72]**
MRT	**.76 [.63, .88]**	**.67 [.54, .79]**			**.10 [.04, .15]**
** **RTV	**.78 [.65, .89]**	**.64 [.53, .76]**			**.14 [.08, .19]**
** **OE	**.70 [.55, .90]**			**.16 [.06, .32]**	**.54 [.38, .68]**
CongE	**.80 [.61, .95]**			**.32 [.16, .52]**	**.48 [.31, .67]**

*Note.* Significant estimates are indicated in bold. ERP = event-related potential; IQ = intelligence quotient; DSF = digit span forward; DSB = digit span backward; ERN = error-related negativity amplitude from the flanker task; NoGo-P3 = P3 amplitude in the NoGo condition from the cued continuous performance test; MRT = mean reaction time from the fast task; RTV = reaction time variability from the fast task; OE = omission errors from the cued continuous performance test; CongE = errors in the congruent condition of the flanker task; Common F1-F3 = the standardized variance of each variable explained by familial and nonfamilial factors. Total = the total standardized variance of each variable due to familial and nonfamilial influences.

The factor structure of nonfamilial influences resembled that of familial influences, except for IQ which loaded on the same factor capturing DSF and DSB (Figure 1). The majority of the nonfamilial variance of most cognitive-ERP measures was not explained by these three factors (cNf_1-3_), but by specific influences, apart from MRT and RTV which were more strongly influenced by a common nonfamilial factor (cNf_1_) (Table 4). The nonfamilial factors showed moderate-to-high intercorrelations and moderate correlations with nonfamilial influences on ADHD. The phenotypic correlation between each cognitive-ERP variable and ADHD was explained to a similar extent by shared familial and nonfamilial factors (Table S5).

## Discussion

This study represents the first comprehensive investigation to date, using a broad range of cognitive-performance and brain activity (EEG) measures, into the etiology underlying cognitive-neurophysiological impairments in ADHD that has persisted from childhood to adolescence and early adulthood. We identified three familial and three nonfamilial factors underlying the association between impairments in these measures and ADHD. The familial factors captured (a) response speed (MRT) and variability (RTV), and IQ; (b) short-term (DSF) and working (DSB) memory; and (c) sustained attention (OE, CongE), error processing (ERN), and, to a smaller extent, response inhibition (NoGo-P3). Familial influences on ADHD overlapped strongly with both the first and third factors, but only moderately with the memory (second) factor. The same number of factors emerged for nonfamilial influences, with the only exception that IQ clustered with memory rather than RT measures. These findings identify multiple partially separable etiological processes that underlie cognitive-neurophysiological impairments in persistent ADHD, extending our understanding of the etiological pathways to widespread cognitive and brain dysfunction in ADHD in adolescence and adulthood.

Our results show substantial shared familial influences between cognitive-neurophysiological impairments and ADHD in adolescents and young adults. The factor model further indicates that the association between these impairments and ADHD (*r*_cF1-ADHD_ =.50; *r*_cF2-ADHD_ = −.36; *r*_cF3-ADHD_ = −.66) may underlie multiple familial processes. The factor structure for familial effects pointed to a separation between a factor capturing IQ and RT performance (cF_1_), a factor capturing memory performance (cF_2_), and a factor capturing accuracy (number of errors) and brain activity of inhibitory/error-detection processes (cF_3_). The separation between factors indicates that the co-occurring presence of impairments captured by the same factor could be largely explained by shared familial influences. For example, the finding that one familial factor captured both IQ and RT performance indicates a strong familial association between these measures (more than with other measures) in adolescents and adults. Conversely, impairments that are captured by two separate factors may be driven by at least partially separate familial pathways. A dissociation of this kind is shown for memory and RT performance, indicating that impairments in these processes may result from partly independent etiological pathways. In addition, to our knowledge, this is the first family model-fitting study that simultaneously investigated multiple cognitive and brain measures to obtain a deeper understanding of ADHD. Our results provide new insights into how cognitive-performance impairments (omission and congruent errors) are etiologically associated with neural processes of error detection (ERN) and response inhibition (NoGo-P3), as these four measures clustered in one factor. As such, the etiological factors underlying atypical brain activity of inhibitory and error-detection processes may overlap with those linked to task accuracy indices of sustained attention deficits. The familial factor capturing these four measures (cF_3_) also overlapped with two thirds of the familial influences on ADHD (*r*_cF3-ADHD_ = −.66), indicating a strong etiological association between this cognitive-EEG factor and the disorder.

More generally, our results point to a multifactorial structure of impairments in cognitive and brain function in ADHD, in line with models on ADHD proposing that cognitive and brain dysfunction in the disorder may arise from multiple pathways (Castellanos & Proal, 2012; Halperin & Schulz, 2006; Johnson, 2012). This multifactorial structure may explain the observed individual differences in cognitive profiles that exist among adolescents and adults with ADHD, who may display various degrees of impairments in different cognitive domains (Mostert et al., 2015). A possible clinical implication of these findings is that future efforts to implement new treatments for ADHD could consider including various intervention components, each targeting these different cognitive processes. Given the partial etiological dissociation between the identified cognitive clusters in ADHD, impairments in these factors may have different roles in relation to ADHD pathophysiology. For example, it may be that only some impairments represent mediators lying on the causal pathways to ADHD, while others may only represent associated characteristics (Kendler & Neale, 2010). This partial dissociation between these processes should be considered in future research efforts aiming to examine the role of these impairments in the pathways to ADHD.

Our study provides new evidence on the etiological processes underlying impairments in cognitive and brain function in ADHD adolescence and adulthood. These findings are largely consistent with two earlier findings in childhood (Frazier-Wood et al., 2012; Kuntsi et al., 2010). First, the separation of the factor capturing RT performance from the factor capturing response-accuracy measures is consistent with the separation between MRT/RTV and omission/commission errors found in a multisite study which included data from the sample of the current study in childhood (Kuntsi et al., 2010). Second, the separation between etiological influences on RT and memory performance in adolescents and young adults is further consistent with another study in children where RTV and working memory were captured by two different factors (Frazier-Wood et al., 2012). Differences between this analysis and previous childhood studies were observed in the extent of the etiological overlap among IQ, RT performance and ADHD. In the present study, IQ and RTV/MRT were captured by a single familial factor (cF_1_) highly correlated with ADHD, suggesting substantial overlap in familial variance between these measures. The previous analyses in childhood, however, found a separation of genetic/familial influences on IQ from influences on ADHD and other cognitive impairments (Rommelse et al., 2008; Wood et al., 2010; Wood et al., 2011), suggesting that IQ may represent a separate process. For example, two studies in children reported that the majority (66%-81%) of the genetic/familial influences on IQ were independent of those shared between RT impairments and ADHD (Wood et al., 2010; Wood et al., 2011). Previous analyses on this sample, however, also indicate that lower IQ, both in childhood and at follow up, predicted ADHD persistence (Cheung et al., 2016; Cheung et al., 2015). As such, one possible explanation for the substantial overlap in familial influences between IQ and ADHD in this older age group is that IQ is a potential moderator of ADHD outcome from childhood to adolescence and adulthood. Future longitudinal analyses are needed to elucidate these developmental associations between ADHD and impairments in cognitive and neural processes throughout the development.

It is of interest to note that the separation of familial factors was similar to the distinct processes underlying ADHD persistence and remission previously reported in phenotypic analyses on this sample (Cheung et al., 2016; Michelini, Kitsune, Cheung, et al., 2016). Specifically, IQ/RT and attention/error-processing measures, here captured by two factors with substantial familial sharing with ADHD, were associated with severity and persistence of ADHD in phenotypic analysis (Cheung et al., 2016; Michelini, Kitsune, Cheung, et al., 2016). One possible prediction from these findings is that, in individuals with persistent ADHD, these two factors may jointly contribute to the severity of ADHD and the presence of cognitive-neurophysiological impairments. Conversely, short-term and working memory (here captured by a familial factor that was only moderately overlapping with ADHD) and the response-inhibition NoGo-P3 (here mostly influenced by specific factors not shared with other variables or ADHD) were not sensitive to ADHD persistence/remission in our previous work, in that impairments in these measures did not distinguish between individuals with persistent and remitted ADHD (Cheung et al., 2016). As such, we can hypothesize that impairments in short-term/working memory and in brain activity of inhibition control may reflect separate enduring processes in ADHD associated with persistence of impairments in cognitive and brain function—regardless of severity of ADHD symptoms and impairment.

Nonfamilial influences on ADHD showed moderate overlap with all three nonfamilial factors. Of note, the common factor cNf_1_ captured almost all of the nonfamilial variance shared between ADHD and RT measures, as limited residual variance was not shared with the disorder. Conversely, the nonfamilial variance of IQ and short-term/working memory (cNf_2_), and of sustained attention and inhibitory/error-detection processes (cNf_3_) was largely measure-specific and not shared with ADHD. Nonfamilial influences include individual-specific environmental factors, representing any differences in the environment between siblings, and may include the effects of any treatment for ADHD. A possible prediction is that nonpharmacological interventions, for example cognitive training, aimed at alleviating ADHD symptoms may be more effective if they target RT rather than memory or response-accuracy processes. This prediction is in line with evidence suggesting that RTV may be more malleable than higher-level processes (Kuntsi, Wood, Van Der Meere, & Asherson, 2009) and may explain the low efficacy of treatments targeting working-memory impairments on ADHD (Cortese et al., 2015).

The comprehensive investigation of impairments in cognitive and brain function, with both cognitive-performance and brain-activity measures, and application of sibling model-fitting analyses in a clinical sample are strengths of the current study. One limitation is that sibling data only allow the investigation of familial and nonfamilial effects, but cannot directly estimate the contribution of genetic factors. However, as previous research suggests a limited role of shared-environmental influences on either ADHD (Burt et al., 2012; Nikolas & Burt, 2010) or cognitive-neurophysiological markers (Anokhin, Golosheykin, & Heath, 2008; Kuntsi et al., 2013), the familial overlap between ADHD and such markers is expected to largely reflect genetic influences. Future twin studies are required to confirm this matter. In addition, the age range was wide in our sample. To allow the inclusion children with combined-type ADHD and their siblings at initial assessment, a wide age range was needed for adequate sample size and power for sibling analyses. This prevented us from examining whether the etiological structure of impairments in ADHD may vary with age, as stratifying the analyses by age would have resulted in small samples for sibling analyses. Yet, as we controlled for age in all analyses, we can rule out that our results are confounded by age effects. Future studies using more restricted age ranges should examine these issues.

In conclusion, by using a multivariate approach on a broad range of cognitive and neurophysiological measures, we have identified, for the first time in adolescents and young adults with ADHD, three partially separable factors that captured substantial familial influences (36%-66%) on ADHD and impairments in cognitive and brain function, extending current knowledge from childhood to later development. The familial processes underlying both slower and more variable RTs and lower IQ in adolescents and young adults with ADHD may be partially distinct from familial influences on memory dysfunction and on impairments in sustained attention and brain activity of inhibitory/error-detection processes. These partially distinct etiological pathways may underlie dysfunctional brain networks which are, in turn, associated with impaired cognition and behavior in the disorder. Future efforts should examine the developmental trajectories of these etiological pathways, and test treatment effects on these partially separate cognitive-neurophysiological factors, which would refine causal models of the disorder and point to sensitive targets for interventions.

## Supplemental Material

Supplement_JAD – Supplemental material for The Etiological Structure of Cognitive-Neurophysiological Impairments in ADHD in Adolescence and Young Adulthood Supplemental material, Supplement_JAD for The Etiological Structure of Cognitive-Neurophysiological Impairments in ADHD in Adolescence and Young Adulthood by Giorgia Michelini, Celeste H. M. Cheung, Viryanaga Kitsune, Daniel Brandeis, Tobias Banaschewski, Gráinne McLoughlin, Philip Asherson, Frühling Rijsdijk and Jonna Kuntsi in Journal of Attention Disorders
