# Supplementary material for: The Etiological Structure of Cognitive-Neurophysiological Impairments in ADHD in Adolescence and Young Adulthood
Source: J Atten Disord. 2018 May 3;25(1):91–104. doi: 10.1177/1087054718771191 (PMC7750664; doi:10.1177/1087054718771191)
Supplement: Supplement_JAD – Supplemental material for The Etiological Structure of Cognitive-Neurophysiological Impairments in ADHD in Adolescence and Young Adulthood [file Supplement_JAD.pdf]

SUPPLEMENTARY MATERIAL

The etiological structure of cognitive-neurophysiological impairments in ADHD in adolescence and young adulthood

Further information on the sample

Participants for this study are members of the Sibling EEG Follow-Up Study (SEFOS) (Cheung et al., 2016; Michelini et al., 2016), which investigated neurophysiological and cognitive impairments in a follow-up sample of ADHD and control sibling pairs. Adolescents and young adults, who had taken part in the UK subsample of the International Multicenter ADHD Genetics (IMAGE) project (Chen et al., 2008; Cheung et al., 2012; Kuntsi et al., 2010) when they were children, were invited to participate in this follow-up assessment. During the initial study, ADHD participants aged between 6 and 17 years were recruited from specialist clinics in the UK from among those who had a clinical diagnosis of DSM-IV combined subtype ADHD during childhood. Childhood ADHD was assessed based on the Parental Account of Childhood symptoms (PACS) (Taylor, Everitt, et al., 1986; Taylor, Schachar, Thorley, & Wieselberg, 1986), a semi-structured, standardised, investigator interview with high inter-rater reliability, to establish the research diagnosis of DSM-IV combined-type ADHD in childhood. Closest-age siblings were also then recruited and assessed for ADHD using the same procedures. A control group, which was initially recruited from primary (ages 6-11 years) and secondary (ages 12-18 years) schools in the UK (Kuntsi et al., 2010), was also invited to take part in this follow up study. The total sample in childhood consisted of 267 participants from ADHD sibling pairs and 258 participants from control sibling pairs (n=525 participants). Exclusion criteria included IQ<70, autism, epilepsy, brain disorders and any genetic or medical disorder associated with externalizing behaviours that might mimic ADHD. All participants were of European Caucasian decent. At follow up, participants were

contacted by telephone and scheduled for a single testing session including clinical, cognitive and EEG assessments. Retention rate at follow-up was 77%.

ERP analysis

ERP measures which showed ADHD-control differences in our previous work on this sample were included in this study, following the same ERP protocol used in our previous analyses showing ADHD-control differences in these tasks (Cheung *et al.*, 2017, Cheung *et al.*, 2016, Michelini *et al.*, 2016). ERPs from the cued performance task (CPT-OX) included the Cue-P3, the CNV and the NoGo-P3 (Cheung *et al.*, 2016). ERP components in this task were measured without applying a pre-stimulus baseline correction in line with previous ERP analyses on the same paradigm (Albrecht *et al.* 2013; McLoughlin *et al.* 2010; Cheung *et al.*, 2016). The Cue-P3 amplitude was measured as the maximum positive peak between 250-600ms following cue trials at Pz. The CNV was analysed as mean amplitudes between 1300 and 1650ms following cues at Cz. The NoGo-P3 amplitude was measured as the maximum positive peak between 250-600 ms following NoGo trials at Cz. In the other tasks characterized by inactive pre-stimulus states or analysed by peak-to-peak ERP measures, pre-stimulus baseline correction was applied prior to extracting ERP peaks. As in our previous work (Michelini et al., 2016), ERPs from the arrow flanker task included the N2, the ERN and the Pe from the incongruent condition only, as an N2 reduction in ADHD compared to neurotypical individuals is only observed in the incongruent condition (but not in the congruent condition) of this task (Albrecht et al., 2008; McLoughlin et al., 2009), and a sufficient number of errors to allow at least 20 ERP segments for robust ERP averaging is made in incongruent trials only (McLoughlin et al., 2009). Baseline correction was applied prior to extracting ERP peaks, using the -300 to -100 ms pre-target (-200 to 0 ms pre-flanker) interval. The N2 amplitude was measured as maximum negative peak at Fz and FCz between 250-450 ms after target onset. The ERN amplitude was defined with a peak-to-peak amplitude approach with respect to the preceding positivity (PNe, -100-50 ms) to obtain a more robust measure of this

component (Albrecht et al., 2008; McLoughlin et al., 2009; Nieuwenhuis, Ridderinkhof, Blom, Band, & Kok, 2001), and was measured at FCz between 0-150 ms. The Pe amplitude was measured as maximum positive peak at CPz between 150-450 ms. In the slow-unrewarded baseline condition of the Fast task, the P3 amplitude (baseline-P3) was measured, following pre-stimulus baseline correction in the interval between -200 and 0 ms, as the area amplitude measure at Pz between 250 and 450 ms (Cheung et al., 2017). CNV, Cue-P3, NoGo-P3, N2 and baseline-P3 were stimulus-locked and measured on correct trials only, while the ERN and Pe were response-locked and measured when an erroneous response was made.

Further information on the exploratory factor analysis (EFA)

Our analysis started with an examination of the correlated factors solution of the Cholesky decomposition, which gives separate correlation matrices for the underlying familial and non-familial influences. On the basis of the familial and non-familial correlation matrices between all 9 cognitive-ERP measures, data were simulated in R for 1000 participants within two EFAs, separately for familial and non-familial influences. EFA approaches give an indication of the underlying factor structure, but no specification of the underlying covariance matrices can be deduced. Factors were extracted using an unweighted least squares estimator approach following previous work (Loken, Hettema, Aggen, & Kendler, 2014). An unweighted least squares estimator was chosen over other extraction methods as it has shown robust as a method of factor analysing ordinal data yielding polychoric correlations (Forero, Maydeu-Olivares, & Gallardo-Pujol, 2009; Lee, Zhang, & Edwards, 2012). Factors with an eigenvalue of greater than 1 (Figure S1) were extracted and rotated using an oblique (oblimin) rotation, which allows correlation between factors. The extracted factor structure and factor loadings (Table S3) were specified separately for familial and non-familial influences in a confirmatory factor model in OpenMx. This confirmatory model was aimed at examining the covariation between the factors capturing the cognitive-ERP measures and ADHD.

Further explanation on constrained correlation bivariate models and variable selection

Using our large cognitive battery, previous phenotypic analyses on this sample found that individuals with ADHD showed atypical profiles, compared to controls, in the following 22 cognitive and ERP variables (Cheung et al., 2017; Cheung et al., 2016; Michelini et al., 2016): commission errors, omission errors, mean reaction time (MRT), reaction time variability (RTV), Cue-P3, NoGo-P3 and CNV from the CPT-OX; number of errors in congruent trails (congruent error) and in incongruent trials (incongruent errors), MRT and RTV in both congruent and incongruent conditions, N2, ERN and Pe from the incongruent condition of the arrow flanker task; MRT, RTV and P3 from the baseline condition of the fast task; IQ and digit span forward and backward. In multivariate analyses, due to constraints in the number of variables that can be included in a multivariate SEM model, it was not possible to include all measures (similar to previous quantitative genetic analyses on neurocognitive data; (Frazier-Wood et al., 2012; Kuntsi et al., 2010; McLoughlin, Palmer, Rijsdijk, & Makeig, 2014)). Since our aim was to identify measures associated with ADHD and that would inform on the underlying familial relationships with the disorder, preliminary analyses were thus carried out to objectively select variables more strongly related to ADHD and that showed evidence of underlying familial influences. We ran constrained correlation bivariate models between ADHD and each of the 22 cognitive-ERP variables (Table S1) extracted from our large cognitive-neurophysiological battery, in order to select variables that had (1) modest-to-large (Cohen, 1988) phenotypic correlation with ADHD (phenotypic correlation above .20) and (2) significant cross-sibling/within-trait sibling correlations, suggesting the influence of familial factors (Table S1). These models give maximum likelihood estimates of correlations between two traits within and across pairs while applying specific constraints. Applied constraints reflect the assumptions of the familial model, i.e. that phenotypic correlations across traits within individuals are the same across siblings and that cross-sibling/cross-trait correlations are independent of sibling order. Given the selected nature of this sample (selection of ADHD probands)

and ADHD modelled as present/absent, we further included constraints reflecting the assumptions of the liability distributions underlying ADHD status: we fixed the sibling correlation for ADHD status to .40 and the threshold on ADHD liability to a z-value of 1.64, corresponding to a population prevalence of 5%. Cognitive-ERP variables were modelled as continuous if their age- and sex- residuals were normally distributed or could be normalised using transformations methods, and included with ADHD status in combined continuous-ordinal bivariate models. In these analyses, a model for the thresholds of ordinal variables in specified along with a model for the means of continuous variables. Cognitive-ERP variables which could not be normalised using any transformation methods were modelled as ordinal using equal-sized categories, and included with ADHD status in bivariate ordinal liability-threshold models, estimating age and sex effects on their mean. Ordinal models and combined continuous-ordinal models were used to derive, respectively, the polychoric and polyserial phenotypic correlations between ADHD and each cognitive-ERP variable, the cross-sibling/within-trait sibling correlation for each cognitive-ERP variable, and the cross-sibling cross-trait sibling correlation between ADHD and each cognitive-ERP variable (Table S1).

Information about the precision of parameter estimates was obtained by likelihood-based confidence intervals (CIs). According to the criteria outlined above, IQ, DSF, DSB; MRT and RTV from the fast task (baseline condition); RTV, OE and NoGo-P3 (Figure S3) from the CPT-OX; and RTV in the congruent and incongruent condition, congruent errors (CongE) and ERN (Figure S2) from the arrow flanker task could be retained for inclusion with ADHD status in the multivariate models, as they met both inclusion criteria. Since all measures of RTV across tasks showed large correlations with one another (r=.45-.76), only RTV from the baseline condition of the fast task was included, as this variable showed the strongest phenotypic correlation with ADHD (Table S1).

Model comparisons

The Akaike information criterion (AIC) and χ^2 difference tests were used to inform on model fit when comparing models. The confirmatory 3-factor model showed a significantly better fit compared to the Cholesky decomposition (p=.08) (Table S4), indicating support for this more parsimonious description of the data.

As a sensitivity test, the 3-factor model was also compared to a 1-factor model. In this 1-factor model, all cognitive-ERP variables were influenced by 1 familial factor and 1 non-familial factor, with correlation paths to the familial and non-familial influences on ADHD, respectively. The 1-factor model provided a significantly worse fit than the 3-factor model (p<.01). This suggests that, although the 3 familial and non-familial factors are inter-correlated, they represent processes that are at least partly separable and cannot be accounted for by a single factor.

Proportion of phenotypic correlation due to familial and non-familial factors

The proportion of phenotypic correlation between ADHD and each cognitive-ERP variable explained by contributions of shared familial and non-familial influences could be further derived from the factor model (note that these phenotypic correlations could be slightly different from those estimated from the saturated Cholesky model in Table 1). For example, the proportion of phenotypic correlation between IQ and ADHD is calculated by two pathways: (1) via linked familial factors: the product of the standardised factor loading of IQ (path from cF1 to IQ), the correlation between cF1 and the ADHD-specific familial factor, and the standardized factor loading of ADHD ($\sqrt{.40}$), [$-.64*.50*\sqrt{.40}=-.20$]; and (2) via linked non-familial factors: [$.43*-.40*\sqrt{.60}=-.13$]. These two pathways sum up to the predicted phenotypic correlation according to this model, and the proportions of familial and non-familial overlap work out to be -.20/-.34=60% and -.13/-.34=40%, respectively. Note that proportions can only be derived if the contributions have the same sign. All correlations were similarly explained by shared familial and non-familial factor influences (Table S5).

Table S1. Results of the constrained correlation sibling model of each variable with ADHD in order to select variables of interest

	r_{Ph} with ADHD	Sibling r	Cross-sib/cross-trait r with ADHD
**CPT-OX**			
**OE**	**0.30 (0.18; 0.42)**	**0.22 (0.03;0.40)**	**0.26 (0.13; 0.39)**
**CE**	**0.14 (0.01; 0.26)**	0.11 (-0.09; 0.30)	0.11 (-0.03; 0.23)
**MRT**	**0.19 (0.09; 0.30)**	**0.25 (0.10; 0.39)**	0.09 (-0.03; 0.20)
**RTV**	**0.28 (0.16; 0.38)**	**0.27 (0.13; 0.40)**	**0.16 (0.05; 0.27)**
**Cue-P3**	-0.16 (-0.27; -0.05)	0.14 (-0.02; 0.28)	0.03 (-0.08; 0.15)
**CNV**	**0.25 (0.13; 0.34)**	0.14 (-0.03; 0.31)	0.08 (-0.04; 0.20)
**NoGo-P3**	**-0.25 (-0.36; -0.14)**	**0.25 (0.10; 0.39)**	**-0.16 (-0.31; -0.04)**
**Fast task**			
**MRT-baseline**	**0.35 (0.24; 0.45)**	**0.32 (0.18; 0.44)**	**0.15 (0.03; 0.26)**
**RTV-baseline**	**0.44 (0.34; 0.54)**	**0.28 (0.13; 0.41)**	**0.19 (0.07; 0.30)**
**P3-baseline**	-0.07 (-0.19; 0.06)	**0.26 (0.09; 0.42)**	-0.03 (-0.16; 0.10)
**Flanker task**			
**CongE**	**0.33 (0.20; 0.43)**	**0.22 (0.04; 0.38)**	**0.13 (0.00; 0.25)**
**IncongE**	**0.22 (0.10; 0.33)**	0.10 (-0.05; 0.25)	**0.14 (0.02; 0.25)**
**MRT-cong**	**0.17 (0.05; 0.28)**	**0.22 (0.06; 0.36)**	0.05 (-0.07; 0.17)
**MRT-incong**	**0.13 (0.01; 0.24)**	**0.24 (0.09; 0.37)**	0.03 (-0.09; 0.16)
**RTV-cong**	**0.35 (0.24; 0.45)**	**0.21 (0.07; 0.34)**	**0.12 (0.01; 0.23)**
**RTV-incong**	**0.35 (0.24; 0.45)**	**0.20 (0.05; 0.33)**	**0.14 (0.03; 0.25)**
**N2**	**0.14 (0.03; 0.26)**	**0.29 (0.14; 0.43)**	0.07 (-0.04; 0.20)
**ERN**	**-0.24 (-0.35; -0.12)**	**0.21 (0.04; 0.36)**	-0.09 (-0.21; 0.03)

**Pe**	**-0.19 (-0.30; -0.06)**	**0.24 (0.08; 0.40)**	-0.06 (-0.19; 0.07)
**IQ and Digit Span**			
**IQ**	**-0.38 (-0.48; -0.27)**	**0.50 (0.39; 0.60)**	**-0.17 (-0.28; -0.06)**
**DSF**	**-0.23 (-0.35; -0.11)**	**0.45 (0.32; 0.56)**	**-0.14 (-0.25; -0.01)**
**DSB**	**-0.30 (-0.41; -0.19)**	**0.30 (0.15; 0.43)**	**-0.22 (-0.33; -0.10)**

Note: significant values are given in bold. Variables included in the multivariate factor analysis are highlighted in grey.

Abbreviations: ADHD, attention deficit hyperactivity disorder; CE, commission errors from the cued continuous performance test; CongE, errors in the congruent condition of the flanker task; CPT-OX, cued continuous performance test; Cross-sib/cross-trait r with ADHD, cross sibling, cross trait correlation between each variable and ADHD; Cue-P3, P3 amplitude in the Cue condition of the cued continuous performance test; DSB, digit span backward; DSF, digit span forward; ERN, error-related negativity amplitude from the flanker task; IQ, intelligence quotient; IncongE, errors in the incongruent condition of the flanker task; MRT, mean reaction time from the cued continuous performance test; MRT-baseline, mean reaction time from the baseline condition of the fast task; MRT-cong, mean reaction time from the congruent condition of the flanker task; MRT-incong, mean reaction time from the incongruent condition of the flanker task; N2, N2 component amplitude from the flanker task; NoGo-P3, P3 amplitude in the NoGo condition from the cued continuous performance test; OE, omission errors from the cued continuous performance test; P3-baseline, P3 amplitude from the baseline condition on the fast task; Pe, error positivity amplitude from the flanker task; r_{Ph} with ADHD, phenotypic correlation with ADHD; RTV, reaction time variability from the cued continuous performance test; RTV-baseline, reaction time variability from the baseline condition of the fast task; RTV-cong, reaction time variability from the congruent condition of the flanker task; RTV-incong, reaction time variability from the incongruent condition of the flanker task; Sibling r, correlation between siblings on each variable.

Table S2. Descriptive statistics for cognitive-neurophysiological measures divided by group, with test for statistical difference

	**ADHD probands (n=87)**	**Unaffected siblings (n=100)**	**Controls (n=169)**	***p***	**ADHD probands vs Controls (*p*)**	**ADHD probands vs Unaffected siblings (*p*)**	**Unaffected siblings vs Controls (*p*)**
**IQ**	96.30 (15.23)	102.85 (14.19)	109.79 (12.45)	**<.001**	**<.001**	**<.01**	**<.001**
**MRT**	626.35 (142.81)	576.46 (125.81)	546.48 (118.90)	**<.001**	**<.001**	**<.001**	**.009**
**RTV**	183.16 (129.66)	127.76 (86.52)	102.75 (82.60)	**<.001**	**<.001**	**<.001**	**<.001**
**OE**	2.86 (4.15)	1.19 (1.88)	0.75 (1.53)	**<.001**	**<.001**	**.02**	**<.001**
**CongE**	9.54 (13.12)	5.14 (5.77)	4.24 (8.27)	**<.001**	**<.001**	**.001**	**.02**
**DSF**	9.27 (2.09)	9.82 (2.14)	10.41 (2.11)	**<.001**	**<.001**	.10	**.01**
**DSB**	6.26 (2.42)	6.79 (2.22)	7.96 (2.62)	**<.001**	**<.001**	.06	**<.001**
**ERN**	7.96 (3.67)	9.76 (4.42)	10.36 (4.73)	**<.001**	**<.001**	**.008**	.24
**NoGo-P3**	6.82 (4.50)	7.92 (3.52)	8.93 (3.69)	**<.001**	**<.001**	**.004**	.26

Notes: Significant differences are indicated in bold. Group differences between ADHD and control participants were reported in previous analyses on this sample (Cheung et al., 2016; Michelini et al., 2016).

Abbreviations: ADHD, attention deficit hyperactivity disorder; CongE, number of errors in the congruent condition of the flanker task; DSB, digit span backward; DSF, digit span forward; ERN, error-related negativity amplitude from the flanker task; IQ, intelligence quotient; MRT, mean reaction time from the fast task; NoGo-P3, P3 amplitude in the NoGo condition from the cued continuous performance test; OE, number of omission errors from the cued continuous performance test; RTV, reaction time variability from the fast task.

Table S3. Loadings of extracted familial and non-familial factors from the EFAs

	**Familial factors**			**Non-familial factors**		
	F1	F2	F3	F1	F2	F3
**% Var**	53.19	16.27	12.10	32.01	15.72	11.91
	**Factor loadings**			**Factor loadings**		
**IQ**	**-0.52**	0.33	0.07	-0.09	**0.61**	0.13
**DSF**	0.03	**0.99**	0.04	.06	**0.55**	-0.09
**DSB**	-0.38	**0.67**	0.03	.02	**0.44**	-0.08
**ERN**	-0.16	-0.24	**0.85**	.07	0.03	**-0.64**
**NGP3**	0.07	0.17	**0.38**	-.06	-0.01	**-0.48**
**MRT**	**0.92**	-0.01	-0.05	**0.96**	-0.04	-0.03
**RTV**	**0.98**	0.06	-0.06	**0.82**	0.01	0.15
**OE**	0.14	-0.17	**-0.66**	0.12	0.06	**0.50**
**CongE**	0.02	0.06	**-0.80**	0.05	-0.22	**0.42**
	**Factor correlations**			**Factor correlations**		
**F1**	1			1		
**F2**	-0.31	1		-0.22	1	
**F3**	-0.52	0.32	1	0.45	-0.38	1

Note: the largest factor loadings for each variable are given in bold.

Abbreviations: % Var, percentage of variance explained by each factor; ADHD, attention deficit hyperactivity disorder; CongE, errors in the congruent condition of the flanker task; DSB, digit span backward; DSF, digit span forward; ERN, error-related negativity amplitude from the flanker task; F1, factor 1; F2, factor 2; F3, factor 3; IQ, intelligence quotient; MRT, mean reaction time from the fast task; NoGo-P3, P3 amplitude in the NoGo condition from the cued continuous performance test; OE,

omission errors from the cued continuous performance test; RTV, reaction time variability from the fast task.

Table S4. Model comparisons

Model	EP	-2LL	df	AIC	BIC	moc	χ^2	df	p
**1. Cholesky**	126	8163.76	3438	1287.75	-10284.47	-	-	-	-
**2. 3 Factor**	**64**	**8242.29**	**3500**	**1242.29**	**-10538.63**	**1**	**78.53**	**62**	**.08**
**3. 1 Factor**	55	8320.92	3509	1302.92	-10508.29	2	78.63	9	<.01

Note: the best fitting model is inidicated in bold.

Abbreviations:AIC, Akaike information criterion; BIC, Bayesian information criterion; EP, number of estimated parameters; -2LL, -2 log likelihood statistic; df, degrees of freedom; moc, model of comparison; χ^2, difference in log likelihood statistic.

Table S5. Proportion of phenotypic correlation between cognitive-ERP variables and ADHD explained by familial and non-familial influences, with 95% confidence intervals in brackets

	**Phenotypic correlation with ADHD**	**Explained by shared familial effects**	**Explained by shared non-familial effects**
**IQ**	-0.33 (-0.44; -0.23)	0.60 (0.35;0.82)	0.40 (0.18;0.65)
**DSF**	-0.25 (-0.35; -0.15)	0.49 (0.15;0.75)	0.51 (0.25;0.86)
**DSB**	-0.27 (-0.37; -0.17)	0.49 (0.13;0.75)	0.51 (0.25;0.87)
**ERN**	-0.26 (-0.33; -0.17)	0.49 (0.22;0.75)	0.51 (0.24;0.78)
**NoGo-P3**	-0.22 (-0.30; -0.13)	0.41 (0.08;0.76)	0.59 (0.24;0.78)
**MRT**	0.37 (0.27; 0.46)	0.40 (0.20;0.62)	0.60 (0.38;0.80)
**RTV**	0.37 (0.26; 0.46)	0.41 (0.20;0.63)	0.59 (0.37;0.80)
**OE**	0.35 (0.23; 0.44)	0.65 (0.38;0.86)	0.35 (0.15;0.62)
**CongE**	0.30 (0.19; 0.39)	0.42 (0.13;0.75)	0.58 (0.25,0.87)

Note: All values are significant.

Abbreviations: ADHD, attention deficit hyperactivity disorder; CongE, errors in the congruent condition of the flanker task; DSB, digit span backward; DSF, digit span forward; ERN, error-related negativity amplitude from the flanker task; IQ, intelligence quotient; MRT, mean reaction time from the fast task; NoGo-P3, P3 amplitude in the NoGo condition from the cued continuous performance test; OE, omission errors from the cued continuous performance test; RTV, reaction time variability from the fast task.

Figure S1. Scree plots of Exploratory Factor Analysis for familial (top half) and non-familial (bottom half) factors

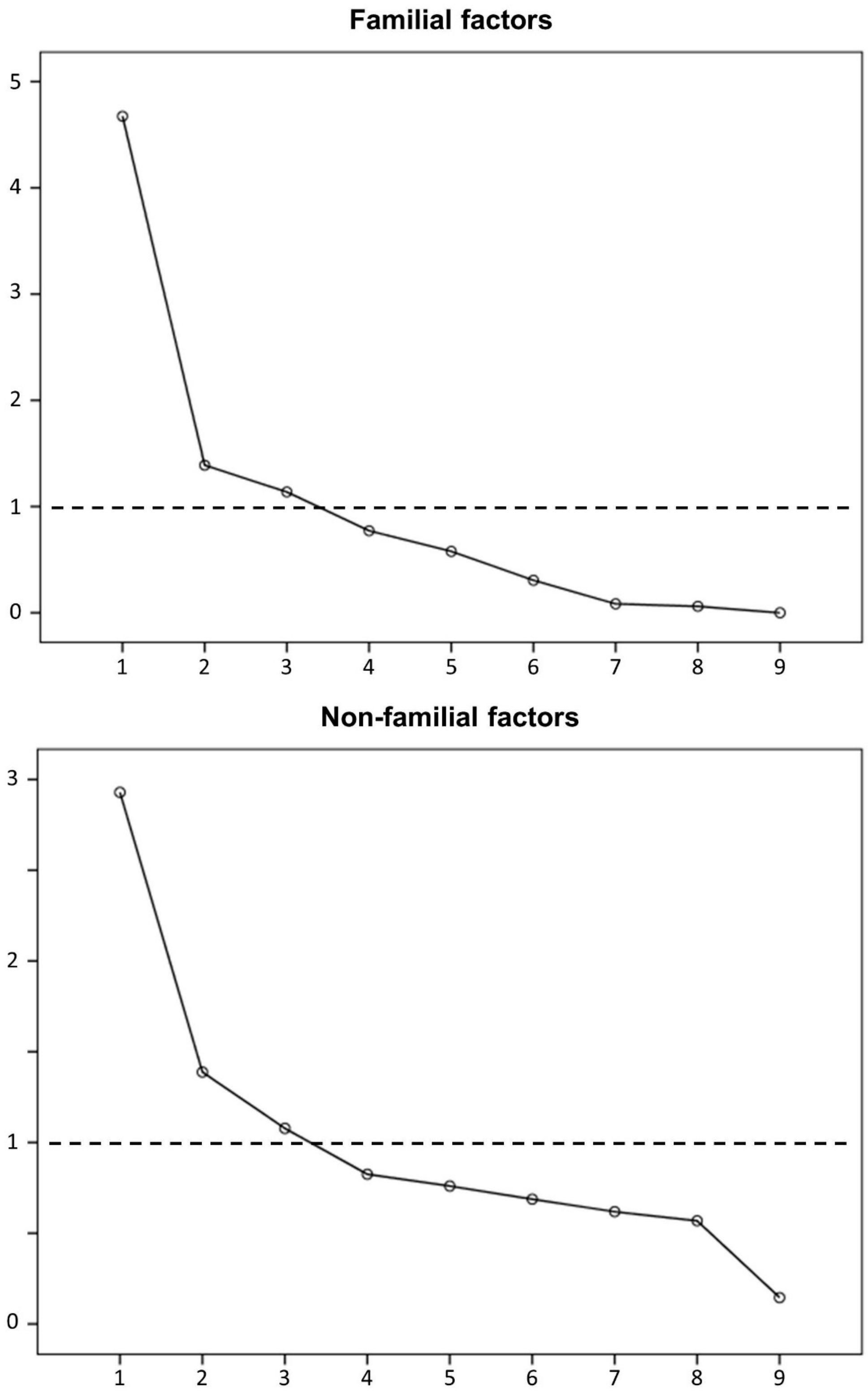

Figure S2. Grand average response-locked ERPs of the ERN at electrode FCz between 0-150 ms after an erroneous response on the incongruent trials for individuals with ADHD (in red), unaffected siblings of ADHD probands (in green) and control participants (in black), with topographic maps.

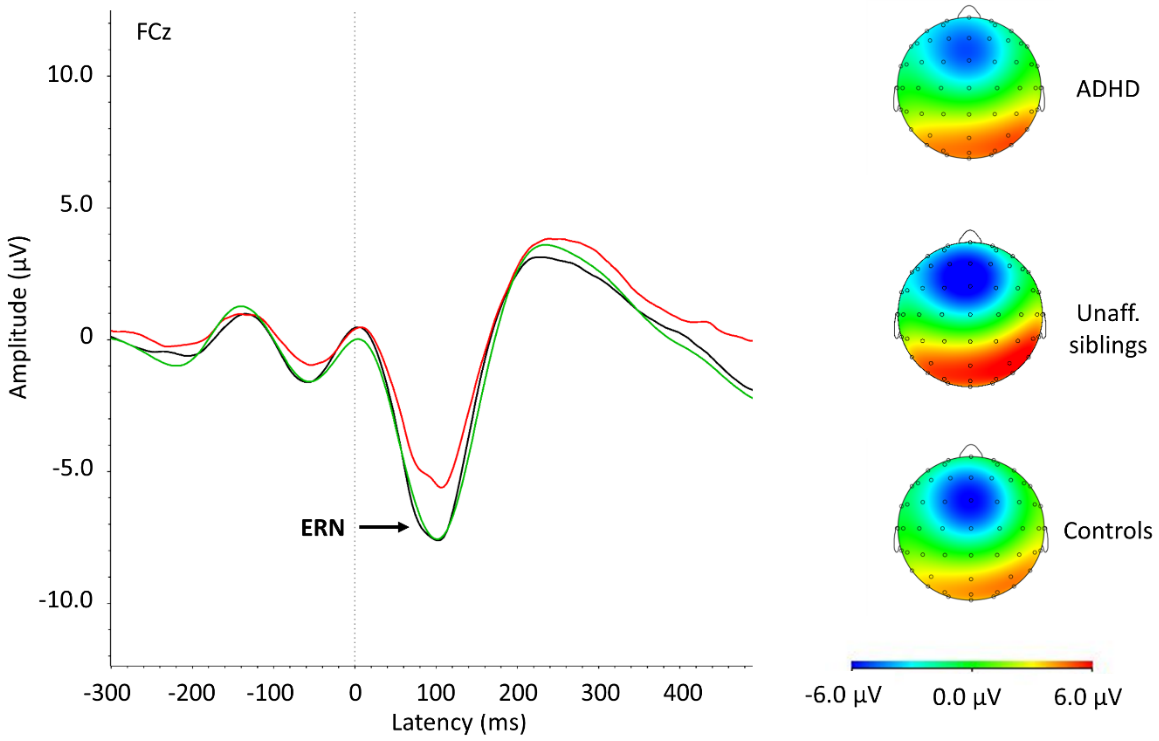

Figure S3. Grand average stimulus-locked ERPs of the NoGo-P3 at electrode Cz between 250-600 ms for individuals with ADHD (in red), unaffected siblings of ADHD probands (in green) and control participants (in black), with topographic maps.

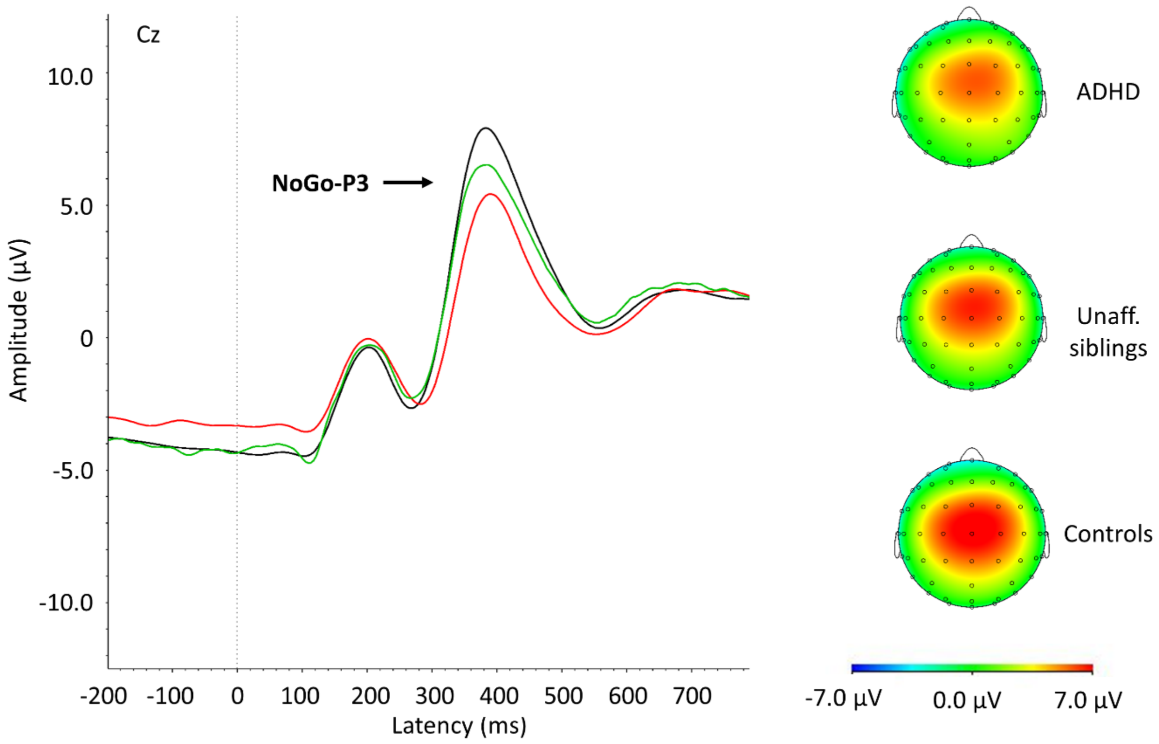

SUPPLEMENTARY REFERENCES

Albrecht, B., Brandeis, D., Uebel, H., Heinrich, H., Mueller, U. C., Hasselhorn, M., . . . Banaschewski, T. (2008). Action monitoring in boys with attention-deficit/hyperactivity disorder, their nonaffected siblings, and normal control subjects: evidence for an endophenotype. *Biol Psychiatry, 64*(7), 615-625. doi:10.1016/j.biopsych.2007.12.016

Chen, W., Zhou, K., Sham, P., Franke, B., Kuntsi, J., Campbell, D., . . . Asherson, P. (2008). DSM-IV combined type ADHD shows familial association with sibling trait scores: a sampling strategy for QTL linkage. *Am J Med Genet B Neuropsychiatr Genet, 147b*(8), 1450-1460. doi:10.1002/ajmg.b.30672

Cheung, C. H., McLoughlin, G., Brandeis, D., Banaschewski, T., Asherson, P., & Kuntsi, J. (2017). Neurophysiological correlates of attentional fluctuation in attention-deficit/hyperactivity disorder. *Brain Topography*.

Cheung, C. H., Rijsdijk, F., McLoughlin, G., Brandeis, D., Banaschewski, T., Asherson, P., & Kuntsi, J. (2016). Cognitive and neurophysiological markers of ADHD persistence and remission. *Br J Psychiatry, 208*(6), 548-555. doi:10.1192/bjp.bp.114.145185

Cheung, C. H., Wood, A. C., Paloyelis, Y., Arias-Vasquez, A., Buitelaar, J. K., Franke, B., . . . Kuntsi, J. (2012). Aetiology for the covariation between combined type ADHD and reading difficulties in a family study: the role of IQ. *J Child Psychol Psychiatry, 53*(8), 864-873. doi:10.1111/j.1469-7610.2012.02527.x

Cohen, J. (1988). *Statistical power analysis for the behavioral sciences* (2nd ed.). Hillsdale, New Jersey: Lawrence Erlbaum Associates.

Forero, C. G., Maydeu-Olivares, A., & Gallardo-Pujol, D. (2009). Factor analysis with ordinal indicators: a Monte Carlo study comparing DWLS and ULS estimation. *Structural Equation Modeling: A Multidisciplinary Journal, 16*(4), 625-641.

Frazier-Wood, A. C., Bralten, J., Arias-Vasquez, A., Luman, M., Ooterlaan, J., Sergeant, J., . . . Rommelse, N. N. (2012). Neuropsychological intra-individual variability explains unique genetic variance of ADHD and shows suggestive linkage to chromosomes 12, 13, and 17. *Am J Med Genet B Neuropsychiatr Genet, 159b*(2), 131-140. doi:10.1002/ajmg.b.32018

Kuntsi, J., Wood, A. C., Rijsdijk, F., Johnson, K. A., Andreou, P., Albrecht, B., . . . Asherson, P. (2010). Separation of cognitive impairments in attention-deficit/hyperactivity disorder into 2 familial factors. *Arch Gen Psychiatry, 67*(11), 1159-1167. doi:10.1001/archgenpsychiatry.2010.139

Lee, C. T., Zhang, G., & Edwards, M. C. (2012). Ordinary Least Squares Estimation of Parameters in Exploratory Factor Analysis With Ordinal Data. *Multivariate Behav Res, 47*(2), 314-339. doi:10.1080/00273171.2012.658340

Loken, E. K., Hettema, J. M., Aggen, S. H., & Kendler, K. S. (2014). The structure of genetic and environmental risk factors for fears and phobias. *Psychol Med, 44*(11), 2375-2384. doi:10.1017/s0033291713003012

McLoughlin, G., Albrecht, B., Banaschewski, T., Rothenberger, A., Brandeis, D., Asherson, P., & Kuntsi, J. (2009). Performance monitoring is altered in adult ADHD: a familial event-related potential investigation. *Neuropsychologia, 47*(14), 3134-3142. doi:10.1016/j.neuropsychologia.2009.07.013

McLoughlin, G., Palmer, J. A., Rijsdijk, F., & Makeig, S. (2014). Genetic overlap between evoked frontocentral theta-band phase variability, reaction time variability, and attention-deficit/hyperactivity disorder symptoms in a twin study. *Biol Psychiatry, 75*(3), 238-247. doi:10.1016/j.biopsych.2013.07.020

Michelini, G., Kitsune, G. L., Cheung, C. H., Brandeis, D., Banaschewski, T., Asherson, P., . . . Kuntsi, J. (2016). Attention-Deficit/Hyperactivity Disorder Remission Is Linked to Better Neurophysiological Error Detection and Attention-Vigilance Processes. *Biol Psychiatry, 80*(12), 923-932. doi:10.1016/j.biopsych.2016.06.021

Nieuwenhuis, S., Ridderinkhof, K. R., Blom, J., Band, G. P., & Kok, A. (2001). Error-related brain potentials are differentially related to awareness of response errors: evidence from an antisaccade task. *Psychophysiology, 38*(5), 752-760.

Taylor, E., Everitt, B., Thorley, G., Schachar, R., Rutter, M., & Wieselberg, M. (1986). Conduct disorder and hyperactivity: II. A cluster analytic approach to the identification of a behavioural syndrome. *Br J Psychiatry, 149*, 768-777.

Taylor, E., Schachar, R., Thorley, G., & Wieselberg, M. (1986). Conduct disorder and hyperactivity: I. Separation of hyperactivity and antisocial conduct in British child psychiatric patients. *Br J Psychiatry, 149*, 760-767.
